# Composition and co-occurrence patterns of the microbiota of different niches of the bovine mammary gland: potential associations with mastitis susceptibility, udder inflammation, and teat-end hyperkeratosis

**DOI:** 10.1186/s42523-020-00028-6

**Published:** 2020-04-14

**Authors:** Hooman Derakhshani, Jan C. Plaizier, Jeroen De Buck, Herman W. Barkema, Ehsan Khafipour

**Affiliations:** 1 Present Address: McMaster University, Faculty of Medicine, Hamilton, ON Canada; 2 Department of Animal Science, University of Manitoba, Winnipeg, MB Canada; 3 Department of Production Animal Health, Faculty of Veterinary Medicine, University of Calgary, Calgary, AB Canada; 4 Present Address: Cargill, Animal Nutrition and Health Division, Cargill Health Technologies, Diamond V brand, Cedar Rapids, IA USA

**Keywords:** Mammary gland, Microbiota, Foundation taxa, Mastitis susceptibility

## Abstract

**Background:**

Within complex microbial ecosystems, microbe-microbe interrelationships play crucial roles in determining functional properties such as metabolic potential, stability and colonization resistance. In dairy cows, microbes inhabiting different ecological niches of the udder may have the potential to interact with mastitis pathogens and therefore modulate susceptibility to intramammary infection. In the present study, we investigated the co-occurrence patterns of bacterial communities within and between different niches of the bovine mammary gland (teat canal vs. milk) in order to identify key bacterial taxa and evaluate their associations with udder health parameters and mastitis susceptibility.

**Results:**

Overall, teat canal microbiota was more diverse, phylogenetically less dispersed, and compositionally distinct from milk microbiota. This, coupled with identification of a large number of bacterial taxa that were exclusive to the teat canal microbiota suggested that the intramammary ecosystem, represented by the milk microbiota, acts as a selective medium that disfavors the growth of certain environmental bacterial lineages. We further observed that the diversity of milk microbiota was negatively correlated with udder inflammation. By performing correlation network analysis, we identified two groups of phylogenetically distinct hub species that were either positively (unclassified Bacteroidaceae and *Phascolarctobacterium*) or negatively (*Sphingobacterium*) correlated with biodiversity metrics of the mammary gland (MG). The latter group of bacteria also showed positive associations with the future incidence of clinical mastitis.

**Conclusions:**

Our results provide novel insights into the composition and structure of bacterial communities inhabiting different niches of the bovine MG. In particular, we identified hub species and candidate foundation taxa that were associated with the inflammatory status of the MG and/or future incidences of clinical mastitis. Further in vitro and in vivo interrogations of MG microbiota can shed light on different mechanisms by which commensal microbiota interact with mastitis pathogens and modulate udder homeostasis.

## Introduction

Milk contains a complex array of bioactive molecules that play fundamental roles in educating the immune system of newborns. Immunoglobulins, lysozymes, lactoferrins, antimicrobial peptides, and oligosaccharides are among immunoregulatory components of milk that act synergistically to maintain the intestinal homeostasis of neonates [1, 2]. In addition, milk also provides a nutrient-rich ecosystem for a diverse range of commensal and pathogenic microorganisms to thrive [3, 4]. These microbes not only serve as important ecological seed species for the developing gut microbiota of neonates, but also interact with the immune system of the mammary gland (MG) [5] and confer modulatory influences on inflammatory responses and susceptibility to infections [6].

In the bovine MG, mastitis is characterized by inflammation in response to metabolic disorders, trauma, and more frequently, intramammary infection (IMI). The latter often occurs upon transgression of opportunistic and obligate pathogens past the teat canal [7], resulting in activation of both innate and adoptive immune systems. Due to their importance to the dairy industry and animal welfare, epidemiology and pathogenesis of major mastitis-causing bacteria (e.g. *Staphylococcus aureus*, *Streptococcus dysgalactiae,* and *Escherichia coli*) have been extensively investigated using a wide range of culture-dependent and/or molecular techniques [8]. It is now well understood that several genetic, physiological, and environmental factors are capable of modulating the defense mechanisms of the MG against each of these pathogens [9]. In addition, commensal microbiota inhabiting different ecological niches of the udder (i.e. teat apex, teat canal, and milk [4]) have the potential to govern susceptibility to IMI by mastitis pathogens via several mechanisms. For instance, certain non-*aureus* staphylococci (NAS) and *Corynebacterium* species colonizing the teat apices and teat canals of dairy cows have the ability to produce a wide range of bacteriocins, and, therefore, prevent growth of major mastitis pathogens [10, 11]. However, microbe-microbe cross talks are inherently complex and those aspects that are crucial to functional properties of the mammary ecosystem, such as resilience to colonization by pathogens, are as yet poorly understood. Within complex ecosystems, certain species play disproportionately large roles in shaping the overall structure and stability of the community. In other words, by promoting beneficial interactions within the community, these “foundation species” can increase the diversity of the ecosystem and make it more resilient against invasion by exogenous species [12, 13]. Thus, identification of potential foundation species within the bacterial ecosystem of the MG can serve as an important step for understanding the mechanisms by which microbiota contribute to mammary homeostasis and susceptibility to IMI by mastitis pathogens.

To date, several studies have explored the global diversity of milk and teat canal microbiota in relation to udder health parameters [14–17]. Besides expected inter-study differences in the compositions of MG microbiota, a common finding among them has been the association of dysbiotic microbiota with the incidence of mastitis. Kuehn et al. [14] reported a distinct clustering pattern between the microbiota of milk samples obtained from healthy quarters and those belonging to culture-negative clinical mastitis (CM) ones. Oikonomou et al. [15, 18] and Ganda et al. [16] also observed that the microbiota of milk samples derived from clinically affected quarters had reduced richness and evenness compared to those obtained from healthy quarters. Despite valuable insights provided by these studies into the compositional differences between the microbiota of healthy and mastitic quarters, the potential role of commensal microbiota in maintaining MG homeostasis and modulating mastitis susceptibility remains largely unknown. In the present study, we explored the bacterial composition of MG quarters from varying levels of inflammation (as determined by somatic cell count (SCC) of milk samples [19]) in order to: a) characterize bacterial communities that inhabit different ecological niches of the MG (teat canal vs. milk), b) determine potential associations of different bacterial taxa with the inflammatory status of the udder, c) characterize niche-specific bacterial co-occurrence patterns in order to identify potential hub species and foundation taxa, and d) determine whether candidate foundation taxa are associated with the biodiversity of the MG and susceptibility to CM.

## Results

### Biodiversity and taxonomic composition of teat canal and milk microbiota

None of the negative controls included in DNA extraction or PCR reactions resulted in visible PCR bands on gel electrophoresis. These samples were further subjected to sequencing; negative controls of PCR reactions yielded less than 100 reads and sequencing reads obtained from negative controls of DNA extraction ranged between 134 and 285 reads per sample (Additional file 2, Table S7). Due to taxonomic overlap among negative control OTUs with OTUs detected in milk and swab samples, removal of these OTUs from the entire dataset was not feasible. However, a filtering threshold of > 4000 reads per sample was applied in order to exclude low biomass samples from downstream analyses and minimize potential effects of contaminant DNA on the microbiota profile of milk and swab samples. De novo clustering of sequences at 97% similarity threshold resulted in identification of 815 (SD = 205) and 510 (SD = 214) representative bacterial OTUs for TC and milk samples, respectively. Firmicutes, Proteobacteria, Bacteroidetes, and Actinobacteria were predominant bacterial phyla in both niches of the MG. Proportion of 20 most abundant non-random OTUs (present in at least 25% of samples) within the microbiota of healthy udder quarters (determined by a SCC < 200,000 cells/mL) are presented in Additional File 1 - Figure S1. The most abundant OTUs within TC microbiota were those belonging to phylum Proteobacteria [including OTU1 (*Cellovibrio*), OTU7 (*Acinetobacter*), OTU2 (*Stenotrophomonas*), and OTU6 (*Comamonas*)], phylum Firmicutes [including OTU3 (Unclassified Bacillales), OTU5 (top BLASTN bit-scores *Staphylococcus xylosus*), and OTU58 (Unclassified Clostridiales)], and phylum Actinobacteria [including OTU8 (*Arthrobacter*)]. With a slightly different profile, abundant OTUs within milk microbiota included members of Proteobacteria [including OTU14 (Enterobacteriaceae), OTU7 (*Acinetobacter*), OTU1 (*Cellovibrio*), OTU424 (*Sphingobium*)*,* and OTU2 (*Stenotrophomonas*)], Firmicutes [including OTU3 (Unclassified Bacillales), and OTU58 (Unclassified Clostridiales)], and Actinobacteria [including OTU8 (*Arthrobacter*)].

**Fig. 1:**
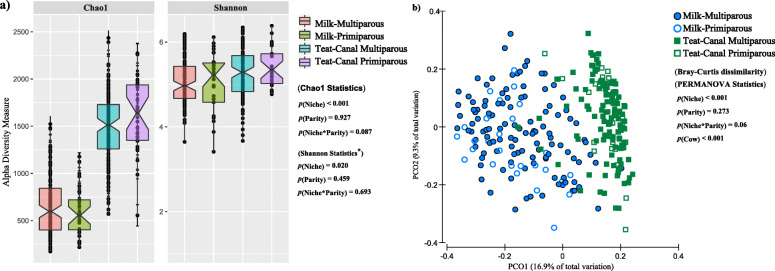
Comparison of diversity metrics between teat canal and milk microbiota. **a** Chao1 index of species richness and Shannon’s index of diversity were compared between the teat canal and milk microbiota of primiparous and multiparous cows. The OTU table was normalized to an even depth of 4000 OTU per sample prior to calculation of diversity metrics. PROC MIXED of SAS 9.3 was used for ANOVA test and the effect of cow was included as random factor in all comparisons. “*” The original values for Shannon’s indices of diversity were subjected to Box-Cox power transformation to achieve normal distribution of the data prior to ANOVA. **b** Principal coordinate analysis (PCoA) was used for visualization of Bray-Curtis dissimilarities of the microbial communities. The OTU table was normalized using cumulative sum scaling (CSS) transformation. PERMANOVA was used to test for distinction of clustering patterns based on different niches of mammary gland and parity. The effect of cow was included as random factor in all comparison. For all tests *p*-values < 0.05 were considered as significant

We next compared the diversity metrics of TC and milk microbiota. Overall, parity did not influence either α- or β-diversity of the MG microbiota at 75 days postpartum. Regardless of parity, microbiota of TC samples were more species-rich (Chao1, *p* < 0.001) and diverse (Shannon, *p* = 0.020) compared to their corresponding milk samples (Fig. 1a). Comparison of Bray-Curtis dissimilarities (Fig. 1b) and weighted UniFrac distances (Additional File 1 - Figure S2) of microbial communities also revealed distinct (*p*_(niche*)*_ < 0.001) clustering patterns between the microbiota of the two niches. Moreover, PERMANOVA analysis revealed a significant (*p*_(cow*)*_ < 0.001) impact of the host animal on shaping the microbiota profile of the MG, with the quarters belonging to the same cow harbouring a more similar bacterial composition compared to their unrelated counterparts.

**Fig. 2:**
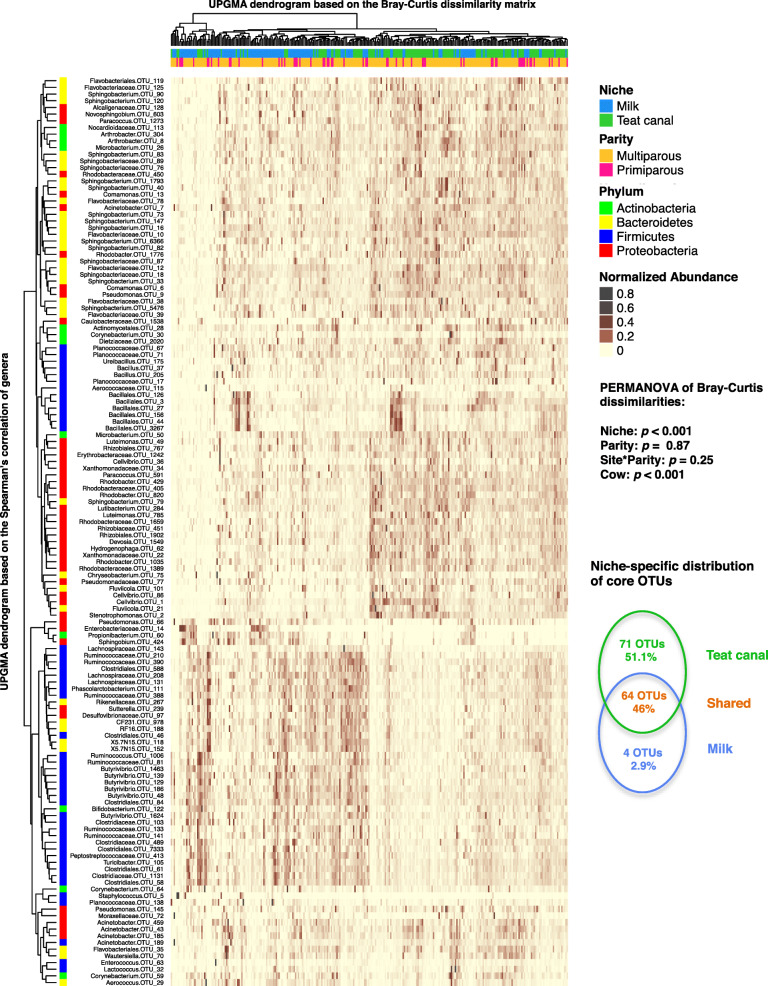
Clustering analysis of mammary gland microbiota based on the distribution of core OTUs. Rows correspond to individual core OTUs (core OTUs defined as those present in at least 75% of samples in each niche and with a relative abundance of > 0.01% of the community). Columns correspond to individual samples. The “Normalized Abundance” key relates colors to the normalized proportions of OTUs (relative abundance of each OTU divided by the Euclidean length of the column vector). The top dendogram shows how samples are clustered based on their Bray–Curtis dissimilarities (using unweighted pair group method with arithmetic averaging (UPGMA)). The significance of clustering patterns has been calculated based on 9999 permutations and *p*-values calculated based on PERMANOVA. The left dendogram shows how OTUs correlate (co-occur) with each other based on their Spearman’s correlation coefficient. The “Phylum” key relates the left annotations to the corresponding phylum of each genus. The “Niche“ and “Parity“ keys relate samples to their originating niche (teat canal vs. milk) and parity group (primiparous vs. multiparous). The VENN diagram shows the distribution of core OTUs within each niche of the mammary gland; “green“ shows the proportion of OTUs that were exclusively core in teat canal microbiota, “blue“ shows the proportion of OTUs that were exclusively core in milk microbiota, and “orange” shows the proportion of OTUs that were identified as core microbiota in both niches

In general, TC microbiota was predominated by OTUs belonging to phyla Proteobacteria and Bacteroidetes, and to a lesser amount comprised of OTUs belonging to Firmicutes and Actinobacteria. On the other hand, the majority of OTUs that were overrepresented in milk samples belonged to the phylum Firmicutes (Fig. 2). OTUs belonging to taxa *Sphingobium, Pseudomonas,* and Enterobacteriaceae (from Proteobacteria)*, Propionibacterium* (from Actinobacteria)*,* Planococcaceae, and *Aerococcus* (from Firmicutes) were significantly overrepresented in milk microbiota, whereas OTUs belonging to taxa *Microbacterium*, Nocardioidaceae, *Corynebacterium* (from Actinobacteria), *Paracoccus*, *Comamonas*, *Stenotrophomonas*, *Cellvibrio* (from Proteobacteria), *Bacillus*, *Ureibacillus*, and Planococcaceae (from Firmicutes) were significantly overrepresented in TC microbiota (see Additional Files 2 - Table S1 for the complete list of OTUs that were overrepresented within TC or milk microbiota).

Next, we compared distributions of core OTUs between the two niches of the MG (core OTUs were defined as those present in at least 75% of samples in each niche and with a relative abundance of > 0.01% of the community). A total of 135 core OTUs were detected within TC microbiota, while the microbiota of milk samples only contained 68 core OTUs. Of these, 71 OTUs were exclusively core in TC microbiota, 4 OTUs were exclusively core in milk microbiota, and 64 OTUs were considered as shared core microbiota between the two niches (Fig. 2). OTUs exclusively found to be core in milk microbiota included OTU14 (Enterobacteriaceae), OTU66 (*Pseudomonas*)*,* OTU424 (*Sphingobium*), and OTU60 (*Propionibacterium*).

### Association of the MG microbiota with teat end hyperkeratosis, SCC, and mastitis

We further explored the association of core OTUs of the MG microbiota with udder health parameters and future incidence of CM within the 90-day post-sampling period. Overall, 15 out of 144 quarters were diagnosed with CM during the 90-day post-sampling period. OTU5476 and OTU6366 (both classified as *Sphingobacterium*) were positively associated with the incidence of CM during the 90-day post-sampling period, whereas OTU978 (unclassified Bacteroidales) was negatively associated with future incidence of CM (Additional File 2 - Table S2). OTU5476, OTU1 (*Cellovibrio*)*,* and OTU205 (*Bacillus*) were positively associated with teat end hyperkeratosis scores (Additional File 2 - Table S3). MG quarters included in this trial belonged to a wide range of inflammatory statuses. Out of 144 quarters, the majority were classified as low SCC (< 200,000 cells/mL; *n* = 125), some as high SCC (> 400,000 cells/mL; *n* = 15) and the rest as medium SCC (200,000–400,000 cells/mL; *n* = 4). Associative analysis between the proportion of core OTUs and SCC (treated as a continuous variable) revealed significant negative associations between SCC and proportions of OTU1549 (*Devosia*), OTU304 (*Arthrobacter*), and OTU13 (*Comamonas*), whereas OTU188 (unclassified Bacteroidales) was the only OTU to be positively associated with SCC (Additional File 2 - Table S4).

In addition, we used Spearman’s correlation coefficient to explore relationships between SCC, teat end hyperkeratosis scores, and diversity metrics of the milk microbiota. SCC showed a significant negative correlation with Simpson’s index of diversity (Spearman’s *rho* = − 0.17, *p* = 0.04) and a positive correlation with Bray-Curtis dissimilarities of the milk microbial communities (Spearman’s *rho* = 0.18, *p* = 0.03). Teat end hyperkeratosis scores were not correlated with either SCC or biodiversity metrics (Additional File 1 - Figure S3).

### Niche-specific microbial co-occurrence patterns: identification of hub OTUs

**Fig. 3:**
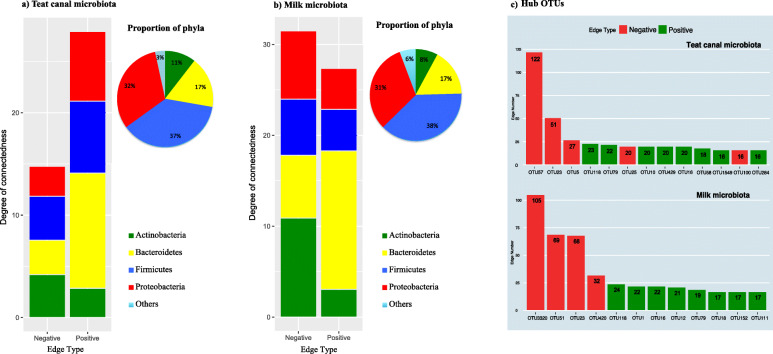
Summary of microbial interaction networks. The degree of connectedness of bacterial phyla has been shown within **a**) teat canal and **b**) milk microbiota. The bar charts show the total number of positive (co-occurrence) or negative (mutual exclusion) interactions observed within the OTUs belonging to major bacterial phyla divided by the average relative abundance of each phylum. The pie charts show the average proportion of bacterial phyla within each niche of the mammary gland. The color codes relate to different bacterial phyla **c**) shows the total number of positive or negative edges (interactions) observed for hub OTUs (OTUs with a minimum number of 16 connections with other members of the microbiota). Color codes denote the type of integrations as revealed by CoNet analysis

Correlation network analysis (CoNet) revealed notable differences between co-occurrence patterns of TC and milk microbiota (Additional File 1 - Figure S4). Overall, the proportion of negative connections (i.e. mutual-exclusion) seemed to be higher within the microbiota of milk samples compared to TC, suggesting that milk may provide a more competitive microbial ecosystem than TC (Fig. 3a and b). Although the proportions of main bacterial phyla were nearly equal within both niches of the MG, their relative degrees of connectedness (total number of positive and negative edges observed for each phylum divided by its relative abundance in the community) varied greatly between the two niches. Within both TC and milk microbiota, Bacteroidetes showed the highest degree of positive connections (i.e. co-occurrence), whereas, Actinobacteria, while constituting a small proportion of the milk microbiota (~ 8% of the community), showed the highest degree of negative connections, suggesting a competitive (inhibitory) role that some members of this bacterial phylum may play within milk ecosystems. Firmicutes and Proteobacteria constituted a large proportion of the microbiota within both niches of the MG (37 and 32%, in teat canal, and 38 and 31% in milk, respectively) while showing relatively moderate degrees of negative and positive connectedness.

In the next step, we identified niche-specific hub OTUs that showed the highest number of positive or negative connections with other members of the community (> 15 connection; Fig. 3c). Within TC microbiota, negatively connected hub OTUs included OTU57 (*Corynebacterium*), OTU23 (*Pseudomonas*), OTU5 (*Staphylococcus xylosus*), and OTU25 (*Staphylococcus chromogenes*). Within milk microbiota, OTU3320 (*Paracoccus*), OTU51 (*Rummeliibacillus*), OTU23 (*Pseudomonas*), and OTU420 (*Corynebacterium*) were the ones with highest number of negative connections. Of note, none of the negatively connected hub OTUs in this study belonged to the phylum Bacteroidetes. Bacteroidetes OTUs such as OTU16 and OTU79 (*Sphingobacterium*), OTU118 (5.7 N15), and OTU10 (Flavobacteriaceae) were all among the hub OTUs with highest number of positive connections within both niches.

Moreover, by exploring the correlation network between TC and milk microbiota, we identified hub OTUs that were central in between-niche microbial interrelationships. Within milk microbiota, *Sphingobacterium* (OTU16 and OTU79), and *Cellvibrio* (OTU1) showed the highest number of connections with TC microbiota. The relative abundances of all of these three OTUs showed a significant negative correlation with α-diversity measures (Shannon’s and Simpson’s indices) of the TC microbiota. On the other hand, within TC microbiota, OTU23 (*Pseudomonas*) and OTU25 (*S. chromogenes*) showed the highest number of negative connections with milk microbiota. The relative abundance of OTU23 was also negatively correlated with richness (Chao1 index) and Bray-Curtis measures of dissimilarities of the milk microbiota (Additional File 1 - Figure S5).

### Identification of candidate foundation taxa within the MG microbiota

**Fig. 4:**
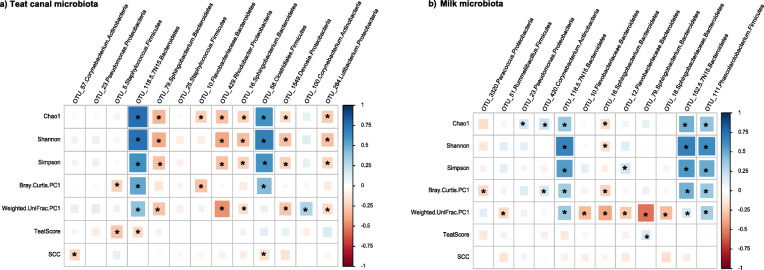
Relationships of hub OTUs with biodiversity metrics of mammary gland and udder health parameters. Spearman’s correlation coefficient was used to explore the relationships between the relative abundances of **a**) teat canal, and **b**) milk hub OTUs and community richness (Chao1 index of richness), α-diversity (Shannon’s and Simpson’s indices of diversity), β-diversity (Bray-Curtis dissimilarities and weighted UniFrac distances of microbial communities) metrics as well as udder health parameters including teat end hyperkeratosis scores and somatic cell counts (SCC) of the milk samples. “*” Indicates *p*-value < 0.05. The color ramp and the size of the squares indicate the type and strength of the Spearman’s correlation coefficient (rho): rho =1 showing strong positive correlation and rho = − 1 showing strong negative correlation between the two parameters

In order to gain deeper insights into influential capacities of hub OTUs, we further examined the relationships (Spearman’s correlation coefficient) of hub OTUs within each niche of the MG with biodiversity metrics, SCC and teat end hyperkeratosis scores. Within TC microbiota, one group of Bacteroidetes OTUs, including those classified as Clostridiales (OTU118 and OTU58) were positively correlated with α- and β-diversity metrics of the microbiota, whereas, another group of Bacteroidetes OTUs, including those classified as *Sphingobacterium* (OTU79 and OTU16) were negatively correlated with diversity metrics. Proteobacterial OTUs, including OTU1549 (*Devosia*), OTU429 (*Rhodobacter*), and OTU284 (*Lutibacterium*) were also negatively correlated with diversity metrics. In addition, OTU57 (*Corynebacterium*) and OTU58 (Clostridiales) were negatively correlated with SCC (Fig. 4a).

**Fig. 5:**
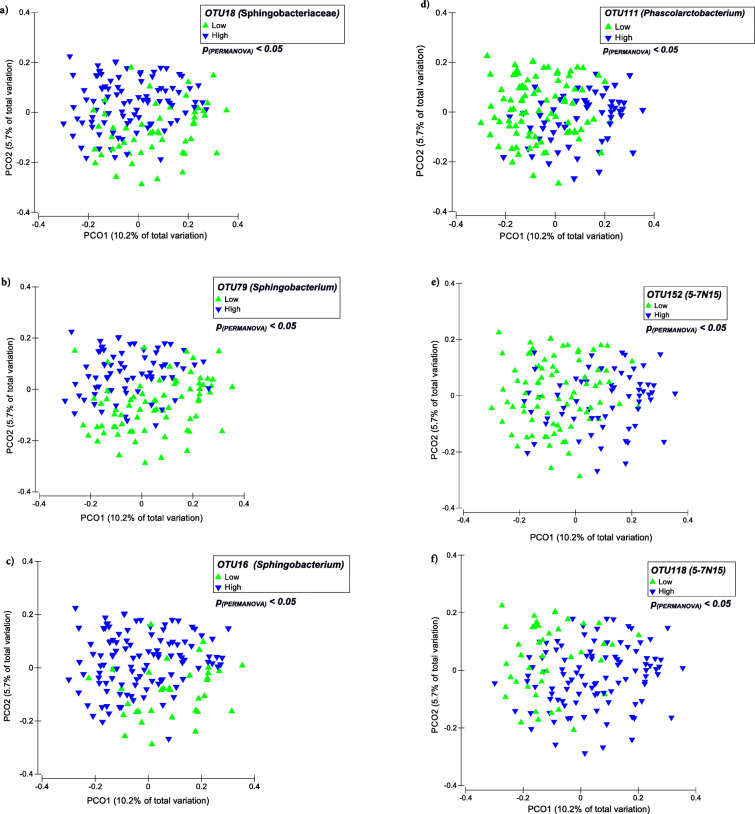
Clustering of milk samples based on the abundance of hub OTUs. Principal coordinate analysis (PCoA) was used for visualization of Bray-Curtis dissimilarities of the milk microbial communities. The OTU table was normalized using cumulative sum scaling (CSS) transformation. PERMANOVA was used to test for distinction of clustering patterns based on the counts of selected hub OTUs (**a-f**). Samples were categorized into two groups that either contained high (≥ 10 OTUs/4000 sequencing reads) or low (< 10 OTUs/4000 sequencing reads) number of the selected hub OTUs. The effect of cow was included as random factor in all comparison. For all tests, *p*-values < 0.05 were considered significant

Within milk microbiota, hub OTUs belonging to Bacteroidetes showed strong associations with biodiversity metrics. Among these, *Sphingobacterium* OTUS including OTU79, OTU16, and OTU18 were negatively correlated with α- and β-diversity metrics of the milk microbiota, whereas Bacteroidaceae OTUs including OTU118 and OTU152 were positively correlated with diversity metrics. In addition, Firmicutes OTU111 (genus *Phascolarctobacterium*) was also positively correlated with diversity metrics of the milk microbiota (Fig. 4b). Categorizing milk samples into two groups that contained either high (≥ 10 OTUs/4000 sequencing reads) or low (< 10 OTUs/4000 sequencing reads) numbers of the above-mentioned hub OTUs resulted in distinct (*p*_(PERMANOVA)_ < 0.05) clustering patterns based on Bray-Curtis measures of dissimilarity (Fig. 5). Comparison of the α-diversity metrics of the milk microbiota based on these categories also revealed significant differences between the two groups; samples containing high abundances of the OTU118, OTU152, and OTU111 had richer and more diverse microbiota compared to those containing lower abundances of these OTUs (Table 1). The latter group of hub OTUs fit the definition of foundation taxa as they are positively connected with other members of the community and appears to be associated with increased ecosystem diversity [12]. No significant difference was observed between the SCC of milk samples containing either high or low abundances of abovementioned hub OTUs.

**Table 1:** Summary statistics comparing biodiversity and somatic cell count (SCC) of milk samples categorized based on high (≥10/4000 OTUs/sample) and low abundances of hub OTUs

OUT ID	SCC				Chao1				Shannon			
	Low^**a**^	High^**b**^	SED^**c**^	***p***-value^**4**^	Low	High	SED	***p***-value	Low	High	SED	***p***-value
**OTU_118**	447,268	172,755	118,920	0.724	487.34	716.52	57.836	< 0.001	6.291	7.460	0.206	< 0.001
**OTU_152**	320,602	162,198	109,986	0.847	533.81	801.67	51.173	< 0.001	6.619	7.776	0.185	< 0.001
**OTU_111**	272,685	222,172	115,742	0.842	557.07	787.77	54.397	< 0.001	6.707	7.725	0.199	< 0.001
**OTU_16**	492,287	180,168	135,939	0.532	661.01	647.19	67.978	0.883	6.524	7.301	0.246	0.046
**OTU_79**	321,900	179,619	115,666	0.299	656.69	643.97	57.319	0.674	6.985	7.258	0.213	0.880
**OTU_18**	402,281	176,670	116,954	0.332	644.31	653.59	59.728	0.595	6.804	7.284	0.219	0.292

^a^< 10/4000 OTUs/sample (OTU table was rarified at even depth of 4000/sample)

^b^≥ 10/4000 OTUs/sample (OTU table was rarified at even depth of 4000/sample)

^c^Standard error of differences of mean squares

^d^Box-cox transformation was performed to achieve normal distribution

### Association of hub OTUs with mastitis susceptibility

By comparing the future incidence of CM between milk samples with either a high or low profile of identified hub OTUs, we observed that in general samples containing high abundances of hub OTUs belonging to *Sphingobacterium,* in particular OTU16, had a higher probability to develop CM during the 90-day post-sampling period. On the other hand, samples that contained high abundances of candidate foundation OTUs (i.e. OTUs that were positively correlated with α-diversity metrics), in particular OTU111, had lower incidence of CM during the same period (Additional File 1 - Figure S6.a). This led us to the speculation that foundation OTUs may, in part, play a modulatory role in the resilience of the MG microbiota against IMI by mastitis pathogens. Therefore, we next explored the relationships between the proportions of hub OTUs and bacterial genera/families that are commonly regarded as mastitis pathogens or opportunists, including *Streptococcus*, *Staphylococcus*, Enterobacteriaceae, *Pseudomonas*, *Corynebacterium*, *Acinetobacter*, and Moraxellaceae. Candidate foundation OTUs (including OTU118, OTU152, and OTU111) showed strong negative correlations with the relative abundances of genera *Pseudomonas*, *Acinetobacter*, and unclassified Enterobacteriaceae. Enterobacteriaceae, along with *Streptococcus*, were the only taxa that showed negative correlations with richness and diversity of milk microbiota. Meanwhile, hub OTUs belonging to *Sphingobacterium* (including OTU16, OTU79, and OTU18) showed positive correlation with genera *Pseudomonas* and *Acinetobacter* (Additional File 1 - Figure S6.b).

**Fig. 6:**
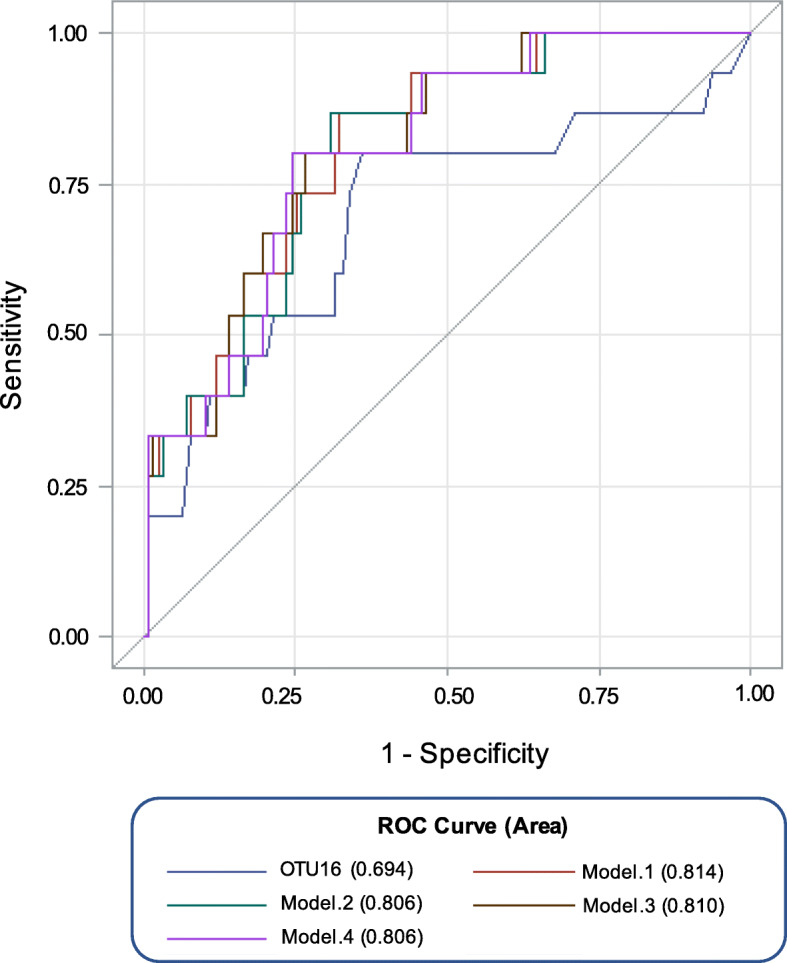
Discriminatory power of selected OTUs for prediction of mastitis susceptibility. Receiver operating characteristics (ROC) curves and area under the curve (AUC) values were used to assess the discriminatory power of the relative abundances of selected OTUs (foundation OTUs and/or OTUs that were associated with the incidence of clinical mastitis during the 90-day post-sampling record keeping period) to predict susceptibility to clinical mastitis. Color codes represent the logistic regression models that were used for ROC analysis: “blue” denotes ROC based on the inclusion of OTU16, “red” denotes model 1: based on the combination of OTU16, OTU6366, and OTU978, “green” model 2: based on the combination of OTU16, OTU6366, OTU111, and OTU978, “brown” model 3: based on the combination of OTU16, OTU6366, OTU79, and OTU978, and “purple” model 4: based on the combination of OTU16, OTU6366, OTU79, OTU978, and OTU 111. The straight line represents the null model

Lastly, the potential of hub OTUs of milk microbiota as predictors of mastitis susceptibility was examined by entering their relative abundances into individual/combination logistic regression models categorized based on the future incidence of CM. OTU16 (*Sphingobacterium*) had the highest discriminative power (AUC = 0.694) in classifying quarters based on future incidence of CM. No other individual OTU, neither from the hub OTUs nor from the ones that MaAsLin identified to be associated with mastitis incidence, could outperform OTU16 with regards to its discriminatory power (see Additional File 2 - Table S5 for summary statistics of the ROC test for all individual OTUs and combination models). However, combined logistic regression models that included OTU16 along with other hub OTUs, particularly those classified as *Sphingobacterium,* were more powerful for prediction of future incidences of CM than the use of OTU16 alone. The highest AUC value was achieved when a combination of *Sphingobacterium* OTUs (OTU16 and OTU6366), and OTU978 (Bacteroidales) were fitted in the model (AUC = 0.814; Fig. 6). In addition, in order to make sure that the discriminatory power of the ROC tests was not affected by the inclusion of the samples that had high SCC at the time of sampling (potentially due to subclinical mastitis), we removed all the samples with SCC > 200,000 cells/mL from the models and repeated the ROC test. Results confirmed similar discriminatory power for all models with only slight drops (< 0.06) in the AUC values compared to original models (Additional File 1 - Figure S7).

## Discussion

Diversity is the central property of an ecosystem that gives rise to other functional properties such as stability, robustness and resilience [20]. In conjunction with environmental and genetic factors, biotic interactions between commensal microbiota have been conceptualized as important driving forces that shape the structure and diversity of microbial communities [21]. In line with this notion, we conducted a cross-sectional study to characterize the core microbiota inhabiting different niches of the bovine MG. Our results provide novel insights into niche-specific microbial relationships and their potential role in shaping the overall structure of the MG microbiota. In addition, we were able to relate the composition and diversity metrics of the MG microbiota to inflammatory status of the udder, underscoring the potential role that this dynamic web of microbes plays in modulating MG homeostasis.

### Structural features of the MG microbiota

Microbial colonization of the teat apex and TC can play principal roles in shaping the intramammary microbiota [22–24]. By performing a comparative analysis between the microbiota of cow’s milk and different environmental sources within dairy systems, others [24, 25] have reported that the microbiota of teat apex and feces were the main contributors to intramammary microbiota. Our results are in general agreement with these reports as we also identified the vast majority of the core OTUs of milk microbiota (> 94%) to be shared with the core microbiota of teat canal, suggesting that intramammary microbiota are in large part shaped by the dispersal of environmental bacteria that colonize or pass through the TC.

Moreover, we observed that the teat canal ecosystem was composed of a more diverse and compositionally distinct microbiota compared to milk. This, coupled with identification of a large number of OTUs that were exclusive to TC microbiota, led us to the proposition that the milk acts as a potent selective medium that precludes the growth of specific bacterial lineages. From an ecological standpoint, the principles of “limiting similarity” [26] suggest that phylogenetic dispersion of microbial communities are driven by strong negative relationships between competing species that tend to thrive on overlapping niches [27]. Our results are in agreement with this hypothesis, in that we observed a high degree of negative connectedness between milk microbiota, which were compositionally and phylogenetically more dispersed compared to teat canal microbiota. Notwithstanding, our network analysis revealed that within each niche of the MG, phylogenetically related OTUs tended to co-occur more often than distant species. For example, as evidenced by our unsupervised clustering analysis, enrichment of a large group of co-occurring gut-associated OTUs, including Ruminococcaceae, Lachnospiraceae, and *Butyrivibrio* (all within the class Clostridia), were the main reason why milk samples clustered distinctly from TC samples. In contrast, TC microbiota were characterized by an overrepresentation of major groups of co-occurring soil-associated OTUs, including *Paracoccus*, Rhizobiales, Rhodobacteraceae, and *Devosia* (all within the class Alphaproteobacteria). Co-occurrence patterns between microbial taxa have been explored in complex microbial communities such as soil [28] and the human gastrointestinal tract [12, 29] to convey information about community assemblage rules. Assuming that phylogeny is closely related to metabolic potential of bacterial species [30], our results suggest that the structure of MG microbiota follows the “habitat filtering” pattern [29] in which phylogenetically related species with similar metabolic capacities tend to co-occur within each niche of the MG. Another possible explanation for the overrepresentation of gut-associated bacteria in milk microbiota would be the “endogenous route hypothesis”, suggesting that cells of the immune system – in particular dendritic cells and macrophages – have the ability to translocate live bacteria from intestinal mucosa to the MG [31]. In ruminants, however, the majority of lymphocytes providing local immunity to the MG originate from peripheral lymph nodes rather than mucosal sites such as intestine [32]. In addition, several other characteristics of the bovine MG immunity, such as the absence of a mucin layer over epithelial cells of the MG and the alertness of mammary epithelial cells and macrophages to sense and respond to microbial antigens via activation of inflammatory cascades, would argue against the possibility of an endogenous route for establishing viable intramammary microbiota [33]. Leaving aside the controversy as to whether or not an endogenous route could be responsible for the development of MG microbiota, our results indicate that microbiota of intramammary secretions share great similarity with microbiota of teat skin and TC. Whether the DNA detected in milk samples originates from viable bacteria, dead cells, or even from bacteria colonizing inside the TC [34], is a debate that has not been settled yet. Future extensive culturomics investigations under anaerobic conditions performed on aseptically collected milk samples might shed light on this aspect of the milk microbiota research.

### Taxonomic composition of the MG microbiota: associations with udder health parameters

Comparing our results with previous studies exploring the global diversity of the teat canal [17, 35], teat apex [36–38], and milk microbiota [14, 15, 18, 39], we identified common bacterial groups that appear to be omnipresent among different niches of the MG. Lactic acid bacteria (LAB; such as *Lactobacillus, Lactococcus,* and *Enterococcus*)*,* psychotrophic bacteria associated with spoilage of milk (such as *Acinetobacter* and *Pseudomonas*), skin-associated bacteria (such as *Staphylococcus* and *Corynebacterium*)*,* and gut-associated Clostridia and Bacilli are among the most frequently identified bacteria within the MG ecosystem across studies. During recent years, non-*aureus* staphylococci (NAS) have gained great attention as leading causes of subclinical mastitis and are omnipresent in the cow’s environment, teat apices, and milk [40, 41]. This group of bacteria is composed of several strains with contradictory functionalities that are either detrimental (i.e. IMI with some NAS species can result in clinical or subclinical mastitis) or beneficial (i.e. some NAS species can provide protection against IMI with major mastitis pathogens) to udder health and milk production [42]. In the present study, none of the identified Staphylococci OTUs were associated with SCC, teat end hyperkeratosis or future incidence of CM. Of note, however, we observed that OTUs belonging to *S. xylosus* and *S. chromogenes* were the most prevalent NAS species within both TC (84.72 and 66.66%, respectively) and milk samples (78.47 and 47.22%, respectively). The persistence and, consequently, prevalence of NAS species in the bovine MG could be in part due to the presence of a wide range of virulence genes that facilitate immune evasion by this group of bacteria and increase their ability to adhere to and colonize mammary epithelial cells [42]. On another note, within the TC microbiota, both *S. xylosus* and *S. chromogenes* were identified as hub OTUs showing a high degree of negative connectedness. A possible explanation for this behavior could be the ability of NAS species to produce a wide range of bacteriocins [10, 43]. However, due to the inability of 16S rRNA sequencing to provide insights into the genetic content and functional properties of bacteria, our study remains inconclusive regarding the true contribution of NAS to shaping MG microbiota and modulating mastitis susceptibility.

Showing a similar trend, *Corynebacterium* (OTU57) was also a hub OTU within TC microbiota that had a high degree of negative connectedness within the community. Several species within the genus *Corynebacterium* have been frequently detected in cow’s milk and associated with IMI [15, 44]. While being considerably less investigated than NAS, Corynebacterial species are also known to produce bacteriocins and exhibit contradictory functionalities with regards to protection against IMI with major mastitis pathogens [11, 45]. Indeed, we observed a negative correlation between the proportion of OTU57 in the TC microbiota and the SCC of corresponding milk samples, suggesting a potential role that this Corynebacterial species might play in modulating the MG homeostasis.

Another group of bacteria that we found to be strongly associated with udder health parameters were the OTUs belonging to *Sphingobacterium.* Oikonomou et al. [15] reported a strong correlation between the proportion of *Sphingobacterium* and increased SCC. Others [46, 47] have also reported the presence of this bacterial lineage in the milk samples obtained from clinical and subclinical cases of mastitis. Our associative analysis also revealed positive links between the proportions of Sphingobacterial OTUs, teat-end hyperkeratosis and future incidences of CM, however, we were unable to find a direct link between them and elevated SCC. Nevertheless, as evidenced by the abovementioned studies, the seemingly wide geographical distribution of the MG-associated *Sphingobacterium* spp*.* warrants further investigations on their potential role as emerging mastitis pathogens worldwide.

### Potential roles of foundation and hub species in modulating community diversity and mastitis susceptibility

Although the link between community diversity and invasibility has been a topic of controversy in macro-ecosystems [48], microbial communities with high species-richness and diversity are believed to be less susceptible to invasion by exogenous perturbants [49]. Positive relationship between species-richness and functional diversity can, but not necessarily [50], give rise to the ecosystem stability: the availability of diverse genomic libraries enables resident microbiota to efficiently use the limiting resources that are available at a given niche and therefore, prevent invader species’ establishment and subsequent growth [20]. In the present work, we focused on biotic interactions that were strongly linked to the biodiversity metrics of the MG microbiota. In particular, we identified certain bacterial taxa, mainly within the phylum Bacteroidetes, which showed strong associations with structure and diversity of the community. Relative to other main bacterial phyla, members of Bacteroidetes encode considerably higher numbers of carbohydrate active enzymes [51], allowing them to be highly flexible in metabolizing glucans from different sources. Therefore, by providing nutrient resources to other members of the community, Bacteroidetes spp. can play a facilitative role in trophic networks and modulate the overall structure of the community (as exemplified in the human gastrointestinal tract [12]). A symbiotic positive feedback, in which species that benefit from the metabolic activity of foundation species in turn facilitate the foundation species, may enhance the overall stability of the ecosystem [13]. In our study, we identified two groups of phylogenetically distinct Bacteroidetes OTUs that were either positively (Bacteroidaceae 5-7 N15) or negatively (*Sphingobacterium*) correlated with biodiversity metrics in both niches of the MG microbiota. This within phylum difference in the behavior of Bacteroidetes spp. is not surprising as even closely related strains within this phylum are known to poses distinct functional properties [52]. Interestingly, the two groups of hub OTUs also behaved differently when it comes to their relationship with bacterial taxa that are associated with mastitis and/or spoilage of milk and dairy products (i.e. Enterobacteriaceae, Acinetobacter and Pseudomonas), suggesting that hub OTUs that are associated with increased diversity of the MG microbiota, may act as potential foundation taxa that can restrict the colonization of pathogenic species.

Dysbiosis and reduced diversity of microbiota have been linked to diseased phenotypes in human and animal models [53]. In bovine MG, Oikonomou et al. [15, 18] reported that the microbiota of samples derived from CM quarters had reduced richness (Chao1 index) and evenness (Shannon index) compared to those obtained from healthy quarters. However, they were inconclusive as to whether dysbiosis was a cause or an effect of the disease. Unfortunately, due to the lack of longitudinal sampling in the present study, we were not able to make direct assessment on the impact of hub OTUs on the stability of the MG microbiota. Nonetheless, the negative links between Sphingobacterial OTUs and biodiversity metrics could be indicative of their potential role in compromising the resistance of MG microbiota against pathogen invasion. Notably, we observed that a high percentage of future cases of CM occurred in the quarters that contained high proportions of Sphingobacterial OTUs. In addition, our logistic regression model revealed a high predictive value for a combination of Sphingobacterial OTUs to discriminate between mastitis-susceptible and resistant quarters. Collectively, these findings suggest that Sphingobacterial-associated dysbiosis of MG microbiota may play a detrimental role in modulation of MG homeostasis and mastitis susceptibility.

It is important to highlight that our study had certain limitations. One of the caveats regarding our results is that microbial relationships are inferred strictly from correlation analyses between the proportions of OTUs. We acknowledge that indirect driving forces such as favorable ecosystem conditions (niche overlap) could also influence co-occurrence patterns within microbial ecosystems. Therefore, associations observed in this study cannot be interpreted as interspecies interactions such as cross-feeding or inhibitory effects due to secretion of inhibitory compounds (e.g. production of bacteriocins). Another caveat of our results is that observed associations are based on the microbiota profiles of TC and milk samples collected from a single dairy farm, and therefore, the results cannot be generalized to other farms across different geographical locations, with different management strategies and different prevalence of IMI by mastitis pathogens. Of particular note is the inherent limitation of 16S rRNA gene sequencing to provide strain-level resolution of the microbiota. Hub OTUs showing strong negative or positive connections in the present study might be represented by different strains in other farms which may not have the characteristics to influence other members of the MG microbiota in a similar fashion. The next limitation of our study is that incidences of CM during the 90-day period after sampling was carried out by visual inspection of clinical signs, and therefore lacked the resolution to identify mastitis pathogens affecting each quarter. This limitation precludes us from evaluating pathogen-specific associations between the profile of mammary microbiota and future incidences of mastitis. Lastly, it is important to recognize the potential effect of DNA contamination of laboratory reagents, in particular DNA isolation kit and PCR reagents, on the microbiota composition of low-biomass samples [54]. Contaminating OTUs detected in laboratory reagents originates from ubiquitous bacterial taxa (e.g. *Corynebacterium*, *Pseudomonas*, *Propionibacterium*, Streptococcus, etc.) [55] many of which are known as native colonizer of the teat skin and MG. Taxonomic overlap among these contaminant OTUs and those from biological samples makes filtering approaches such as removal from the entire dataset impractical. Indeed, contaminant OTUs detected in negative control samples can themselves result from cross-contamination by DNA from samples in neighboring wells during metabarcoding and PCR reaction [56]; suggesting that their removal from the entire dataset can lead to loss of biologically relevant information. In the present study, sequencing of negative control samples resulted in a negligible number of reads compared to actual samples. Therefore, in order to minimize the potential effect of contaminant OTUs on the microbiota profile of mammary gland, we decided to a) exclude low biomass samples from downstream analyses, and b) filter out non-core OTUs with low relative abundance across all samples. Nonetheless, we still recognize the potential existence of contaminant OTUs as a limitation of our study.

## Conclusions

The present study provides novel insights into the structure and interrelationships of the microbiota of different niches of the mammary gland. We observed that TC and milk harbor microbial communities that are phylogenetically distinct from each other. Although TC serves as the main transmission route of exogenous microbiota into the intramammary ecosystem, our data suggest that milk ecosystem precludes the growth of certain environmental bacterial lineages. Furthermore, by investigating within and between niche interrelationships of microbiota we identified hub species that were strongly associated with the diversity of the MG microbiota. In particular, we identified hub species and candidate foundation taxa that were associated with the SCC of the milk and/or future incidence of clinical mastitis, suggesting that they may serve as potential modulators of MG homeostasis and mastitis susceptibility.

## Materials and methods

### Animal selection criteria, teat end hyperkeratosis scoring and herd record-keeping

Quarter milk and teat canal swab samples were obtained from a 500-cow dairy farm in Manitoba, Canada, during the period of December 2014 to February 2015. The sampling protocol was reviewed and approved by the Animal Care Committee of University of Manitoba (protocol number F14–027). All cows recruited for the sampling procedure of this study were housed in a free stall barn that was dedicated to early- and mid-lactation cows. Free stall pens were covered by recycled bedding material renewed every 48 h throughout the study. Bedding material was prepared on-farm by recycling manure solid particles via a bedding recovery unit followed by composting at temperatures > 60 °C. Cows were milked three times a day at 04:00, 12:00 and 20:00 with bedding material being applied after the morning milking. A total of 44 cows (including 9 primiparous and 35 multiparous) at approximately 75 days postpartum were selected and gradually entered the sampling procedure of this study based on the following inclusion criteria: no CM and/or antibiotic therapy during the ongoing lactation, and presence of four functional quarters with no visible sign of CM at the time of sampling (no clotting or abnormal appearance of milk, no swelling or redness of udder). Prior to sampling, hyperkeratosis of teat ends was visually examined and scored as follows: “1” for teat ends with no observable callous ring, “2” for teats ends with a smooth callous ring around the teat orifice, “3” for teat ends with a rough callous ring, and “4” for teat ends with a very rough ring [57]. The farmer was asked to record all incidences of CM for each quarter until 90 days post-sampling. On-farm detection of clinical mastitis was performed by trained milking staff. Udders were examined for clinical signs of mastitis (i.e. swelling, redness, and/or pain) and the foremilk from all quarters were stripped on dark floor mats of the milking parlor for inspection of milk appearance. Cows with inflamed udder and/or quarters showing abnormal milk appearance (i.e. watery appearance, abnormal color, or presence of blood, flakes or clots) were isolated from the herd and subjected to treatment per recommendations of the farm’s veterinarian.

### Sample collection

Samples were collected during the noon milking. TC swab samples (a total of 176 samples from 44 cows) were collected using Ultrafine HydraFlock fiber-tipped swabs (Puritan, Guilford, ME, USA). Prior to sampling, teat ends were thoroughly scrubbed with cotton pads moistened in 70% isopropyl alcohol. The swab was inserted approximately 5 mm into the distal end of the TC and rotated 360°. The swab tip was then aseptically broken and placed into a sterile transport tube containing 1 mL Liquid Amies sterile medium (Puritan, Guilford, ME, USA). This procedure was repeated to obtain a second swab sample from each teat end in order to increase final DNA yield after extraction. Transport tubes were then placed on ice until transfer to the laboratory and stored at − 80 °C. Corresponding quarter milk samples (a total of 176 samples from 44 cows) were collected aseptically following standard recommendations of the National Mastitis Council [58]. In brief, pre-milking teat disinfection was performed using 0.5% iodine pre-dip solution, and teats were thoroughly dried using individual paper towels and then scrubbed for 15 s using cotton pads moistened in 70% isopropyl alcohol. Milk samples (~ 40 mL) were then collected into sterile containers and placed on ice until transfer to the laboratory and aliquoted. One 30 mL aliquot from each sample was used for SCC analysis, performed at Horizon Lab Ltd. (Winnipeg, MB, Canada) using a Fossomatic cell counter (Foss Electric, Hillerød, Denmark). Another two aliquots of 1.5 and 4 mL were stored at − 80 °C until processed for microbial analysis.

### DNA extraction from swab and milk samples

Genomic DNA from TC swab samples was extracted using ZR-96 well Fungal/Bacterial DNA Kit (Zymo Research, Irvine, CA) following modified protocols of the manufacturer as follows: tubes containing swabs and transport medium were defrosted at 4 °C for 4 h, vortexed for 2 min, and centrifuged at 12,000 x g for 15 min. Supernatants were removed and pellets and swab tips were resuspended by adding 200 μL of PBS buffer and vortexing for 30 s. Next, 1 g of autoclaved 0.5 mm silica beads, 400 μL of Lysis Solution (Zymo Research), and 18 μL of 20 mg/mL Proteinase K (Zymo Research) were added to each tube, vortexed for 2 min using a 2010 GenoGrinder (SPEX SamplePrep, Metuchen, NJ) at 1700 strokes per min and subsequently incubated in a heated shaker at 45 °C for 45 min. 400 μL of the resulting mixture was then transferred to the deep-well plate of the Fungal/Bacterial DNA Kit and the extraction process continued following manufacturer’s protocol. Milk samples were processed as follows: 1.5 mL of milk samples were centrifuged at 12,000 x g for 20 min at 4 °C. Supernatants were carefully removed and 200 μL of TE buffer and 300 μL of 0.5 M EDTA (pH = 8) were added to the pellet. The mixture was incubated for 20 min at room temperature and again centrifuged at 12,000 x g for 10 min. Supernatants were removed and pellets were resuspended by adding 200 μL of PBS buffer and vortexing for 30 s. Genomic DNA extraction from the resuspended pellets was then continued similar to the protocol described for swab samples. Negative controls (*n* = 3) were included in both swab (using sterile swabs and transportation medium) and milk (using 1 mL of DNA-free sterile water; ThermoFisher Scientific, Burlington, ON, Canada) extraction protocols.

### PCR amplification and construction of sequencing libraries

The PCR was targeted to amplify V1-V2 regions of the bacterial 16S rRNA genes using modified F27/R357 primers (see Additional File 2 - Table S6) for the complete list of primers used for PCR amplification and sequencing reactions). The forward PCR primer was indexed with 12-base Golay barcodes, allowing for multiplexing of samples. For each sample, PCR reaction was performed in duplicate and contained 3.0 μL of extracted genomic DNA, 1.0 μL of each forward and reverse primer (5 μM), 0.4 μL of 20 mg/mL BSA (ThermoFisher Scientific), 11.6 μL nuclease-free water (ThermoFisher Scientific), and 10 μL of 5 Prime Hot MasterMix (5 Prime Inc., Gaithersburg, MD, USA). Reactions consisted of an initial denaturing step at 94 °C for 3 min followed by 32 amplification cycles at 94 °C for 30 s, 55 °C for 20 s, and 72 °C for 20 s, with a final extension step at 72 °C for 5 min in an Eppendorf Mastercycler pro (Eppendorf, Hamburg, Germany). The sequencing library was then generated as explained by Derakhshani et al. [59] and sequenced using a MiSeq Reagent Kit V3 (600-cycle; Illumina, San Diego, CA, USA) at the Gut Microbiome and Large Animal Biosecurity Laboratories, Department of Animal Science, University of Manitoba, Winnipeg, MB, Canada.

### Bioinformatics and statistical analyses

Default settings of FLASH assembler ver. 1.2.11 [60] were used to merge the overlapping paired-end Illumina fastq files. UPARSE algorithm [61] was used for a) quality filtering of the reads based on maximum expected error value = 1.0, b) identification of unique sequences, c) abundance sorting and removal of singletons, d) clustering the reads into operational taxonomic units (OTUs) based on 97% identity threshold, e) de novo and reference-based chimera checking (against GOLD database [20]), and f) construction of OTU table. Taxonomic classification was then carried out using QIIME [62] implementation of UCLUST (version = 1.2.22) [63] and aligned against the Greengenes database (release May 2013) using the PyNAST algorithm [64]. Phylogenetic trees were built with FastTree ver. 2.1.3 [65]. for further comparison between microbial communities.

Prior to performing downstream analyses, the resulting OTU table was filtered to remove all samples with low sequencing depths (< 4000 sequences per sample) and to keep those that had representative samples from both milk and teat canal of each udder (144 milk samples and their corresponding 144 teat canal swab samples). Community richness (Chao1) and diversity (Shannon) indices were then calculated using QIIME default scripts at an even depth of 4000 sequences per sample. Phylogenetic (weighted UniFrac distances) and abundance-based (Bray-Curtis dissimilarity) β-diversity metrics were calculated following normalization of the final OTU table using the cumulative sum scaling (CSS) transformation [66]. Principal coordinate analysis (PCoA) was applied on the resulting distance matrices to generate two-dimensional plots using default settings of the PRIMER-E software ver. 6.1.18 [67].

Unsupervised clustering analysis was performed to relate clustering patterns of samples to the proportion of core OTUs within each niche of the MG (these OTUs were defined as those present in at least 75% of samples in each niche and with a relative abundance of > 0.01% of the community). Relative abundances of the selected OTUs were normalized across samples (values divided by the Euclidean length of the row vector). Bray–Curtis dissimilarities were calculated using R “vegan” package [68] and the resulting matrix was subjected to unsupervised hierarchical clustering using R “dendextend” package [69] and visualized over a heatmap of the abundance matrix using R “complexheatmap” package [70].

The UNIVARIATE procedure of SAS (SAS 9.3, 2012) was used for testing the normality of residuals for α-diversity measurements. Non-normally distributed data were either log or Box-Cox power transformed and then subjected to an analysis of variance (ANOVA) test using MIXED procedure of SAS. All pairwise comparisons among the groups were tested using the Tukey studentized range adjustment. Permutational multivariate analysis of variance (PERMANOVA; implemented in Primer6 software) was used to detect significant differences between β-diversity metrics of microbial communities. Label permutations (*n* = 9999) were used in PERMANOVA to estimate the distribution of test statistics under the null hypothesis that within-group UniFrac or Bray-Curtis measures are not significantly different from between-group ones. For both ANOVA and PERMANOVA tests, the effect of the different niches of the MG (teat canal versus milk), parity (primiparous versus multiparous), and the interaction between niche and parity were treated as fixed factors whereas the effect of individual animals on quarter microbiota was treated as a random factor.

The relative abundances of selected core OTUs were tested for statistically significant associations with available metadata using multivariate analysis with linear modeling (MaAsLin) [71]. MaAsLin provides the benefit of accounting for all potential confounders (covariates) that could be associated with the profile of microbiota (parity (multiparous vs. primiparous), niche (milk vs. teat canal), teat end hyperkeratosis score, SCC, incidence of CM, and cow (treated as a random factor)). In this approach, a multivariate linear model that associates all available metadata with the relative abundances of OTUs is boosted independently for each OTU. Boosting is used to select metadata that show potential to be associated with OTU abundances. Selected metadata are then used in a general linear model using metadata as predictors and arcsin-square root transformed abundances of OTUs as the response. The multiple hypotheses tested over all OTUs and metadata were adjusted by Benjamini and Hockberg false discovery rate (FDR). For the purpose of this study, significant associations were considered below a q-value threshold of 0.10.

Correlation network analysis (CoNet, [72]) was used to explore microbial co-occurrence/mutual-exclusion relationships and identify hub OTUs that show the highest number of positive/negative correlations with other OTUs. In this ensemble method, a combination of diverse measures of correlation (including Pearson’s, Spearman’s, and Kendall’s correlation coefficients) and dissimilarity (Bray-Curtis, Kullback-Leibler, and Jensen Shannon dissimilarities) were used to overcome major challenges in the inference of co-occurrence networks, particularly those introduced by sparse (zero-inflated) count data, compositionality, and determination of statistical significance. In brief, for each measure, distributions of all pair-wise scores between the nodes (a node representing the relative abundance of a non-singleton OTU that was found in at least 25% of the samples) were computed. For each measure and edge (an edge representing a positive or negative correlation between two nodes), 1000 permutation was conducted (this included a renormalization step for Pearson and Spearman measures in order to address the issue of compositionality introduced by different sequencing depths for each sample). For within niche microbial interactions, the measure-specific *p*-values were then computed as the probability of the null value (represented by the mean of the null distribution) under a Gauss curve generated from the mean and standard deviation of the bootstrap distribution. Measure-specific *p*-values were then merged using Simes’ method [73], and after applying Benjamini–Hochberg’s false discovery rate (FDR) correction, only edges with merged *p*-values < 0.05 were kept. Edges with scores outside the 95% confidence interval defined by the bootstrap distribution and not supported by at least two measures were discarded as well. For between niche microbial interactions, due to the differential distribution of OTUs between the two niches of the MG, bootstrap distribution was not applied and only edges supported by at least three measures were kept in the final network.

The degree of connectedness, a measure used to examine the influential capacity of bacterial taxa [12], was explored at the phylum level by dividing the total number of positive and negative edges observed for each phylum by its relative abundance in the community. In addition, Spearman’s rank correlation coefficient (rho) was used to explore the relationships between hub OTUs within each niche (defined as OTUs with > 15 connections (edges) to other OTUs), biodiversity (α- and β-diversity metrics) and udder health parameters (SCC and teat-end hyperkeratosis scores). Resulting correlation matrices were visualized in heatmaps generated by the Corrplot package of R [74]. Finally, the relative abundances of selected hub OTUs (those showing significant correlations to biodiversity measures) and OTUs that were associated with the incidence of clinical mastitis following the 90-day post-sampling period (as revealed by MaAsLin) were entered into different logistic regression models and their individual/combination potential as predictors of mastitis susceptibility were evaluated using area under the receiver operating characteristics (ROC) curve [75].

## Additional Files

**Additional File 1: Figure S1** Niche-specific distributions of abundant OTUs. **Figure S2** β-diversity comparison of teat canal and milk microbiota. **Figure S3** Relationships between udder health parameters and diversity metrics of the milk microbiota. **Figure S4** Bacterial co-occurrence and co-exclusion networks. **Figure S5** Between-niche relationships of hub OTUs with diversity metrics of microbiota and udder health parameters. **Figure S6** Association of hub species with future incidence of clinical mastitis and proportions of potentially pathogenic bacterial genera. **Figure S7** Discriminatory power of selected OTUs for prediction of mastitis susceptibility.

**Additional File 2: Table S1** Associations of OTUs with deferent niches of the mammary gland. **Table S2.** Associations of OTUs with teat-end hyperkeratosis. **Table S3** Associations of OTUs with future incidence of clinical mastitis. **Table S4** Associations of OTUs with somatic cell count. **Table S5** Summary of statistical analyses (receiver operating characteristic and logistic regression models) for testing the discriminatory power of selected OTUs for prediction of mastitis susceptibility. **Table S6** Sequences of primer set used for barcoded PCR amplification of the V1-V2 regions of the bacterial 16S rRNA genes. **Table S7.** Metadata used to process sequences and perform statistical analyses.

## Data Availability

The sequencing data were deposited into the Sequence Read Archive (SRA) of NCBI (http://www.ncbi.nlm.nih.gov/sra) and can be accessed via accession number SRR6951382. Metadata used to process sequences and perform statistical analyses are presented in Additional File 2 - Table S7.

## Abbreviations

ANOVA: Analysis of variance
AUC: Area under the curve
CoNet: Correlation network analysis
MG: Mammary gland
IMI: Intramammary infection
NAS: Non-*aureus* staphylococci
CM: Clinical mastitis
SCC: Somatic cell count
TC: Teat canal
TE: Tris-EDTA
EDTA: Ethylenediamine tetraacetic acid
rRNA: Ribosomal RNA
PCR: Polymerase chain reaction
FLASH: Fast length adjustment of short reads
OTU: Operational taxonomic unit
QIIME: Quantitative insights into microbial ecology
PyNAST: Python implementation of the nearest alignment space termination
CSS: Cumulative sum scaling
PCoA: Principal coordinate analysis
PERMANOVA: Permutational multivariate analysis of variance
MaAsLin: Multivariate analysis with linear modeling
FDR: False discovery rate
ROC: Receiver operating characteristics curve
SD: Standard deviation
