# Supplementary material for: Composition and co-occurrence patterns of the microbiota of different niches of the bovine mammary gland: potential associations with mastitis susceptibility, udder inflammation, and teat-end hyperkeratosis
Source: Anim Microbiome. 2020 Apr 14;2:11. doi: 10.1186/s42523-020-00028-6 (PMC7807822; doi:10.1186/s42523-020-00028-6)
Supplement: Supplementary file 1 — Additional File 1: Figure S1 Niche-specific distributions of abundant OTUs. Figure S2 β-diversity comparison of teat canal and milk microbiota. Figure S3 Relationships between udder health parameters and diversity metrics of the milk microbiota. Figure S4 Bacterial co-occurrence and co-exclusion networks. Figure S5 Between-niche relationships of hub OTUs with diversity metrics of microbiota and udder health parameters. Figure S6 Association of hub species with future incidence of clinical mastitis and proportions of potentially pathogenic bacterial genera. Figure S7 Discriminatory power of selected OTUs for prediction of mastitis susceptibility. [file 42523_2020_28_MOESM1_ESM.pdf]

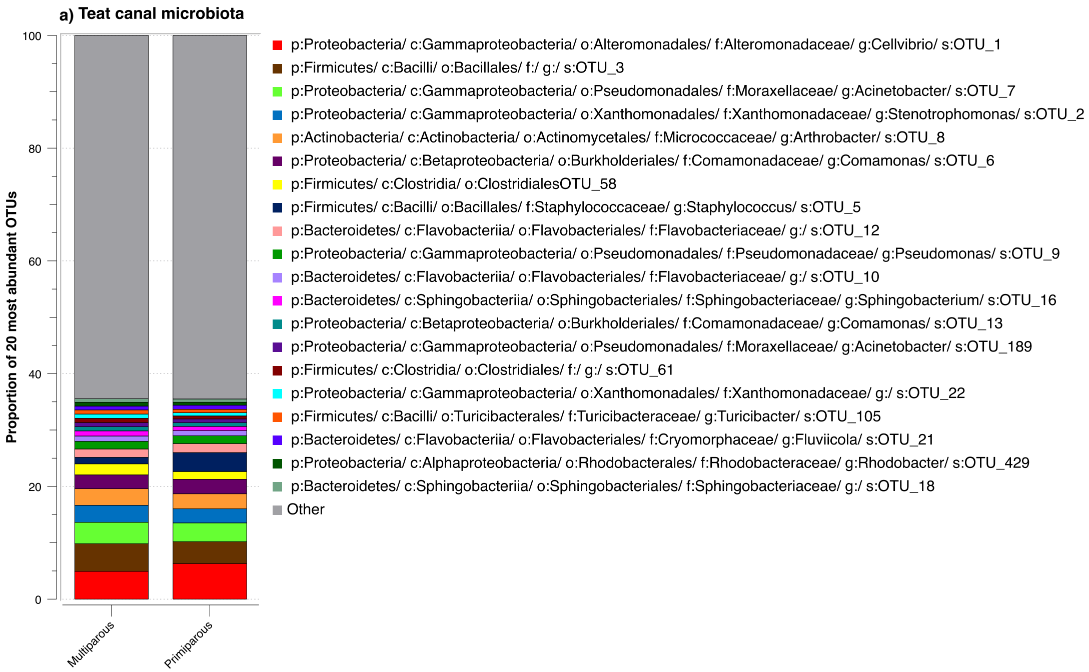

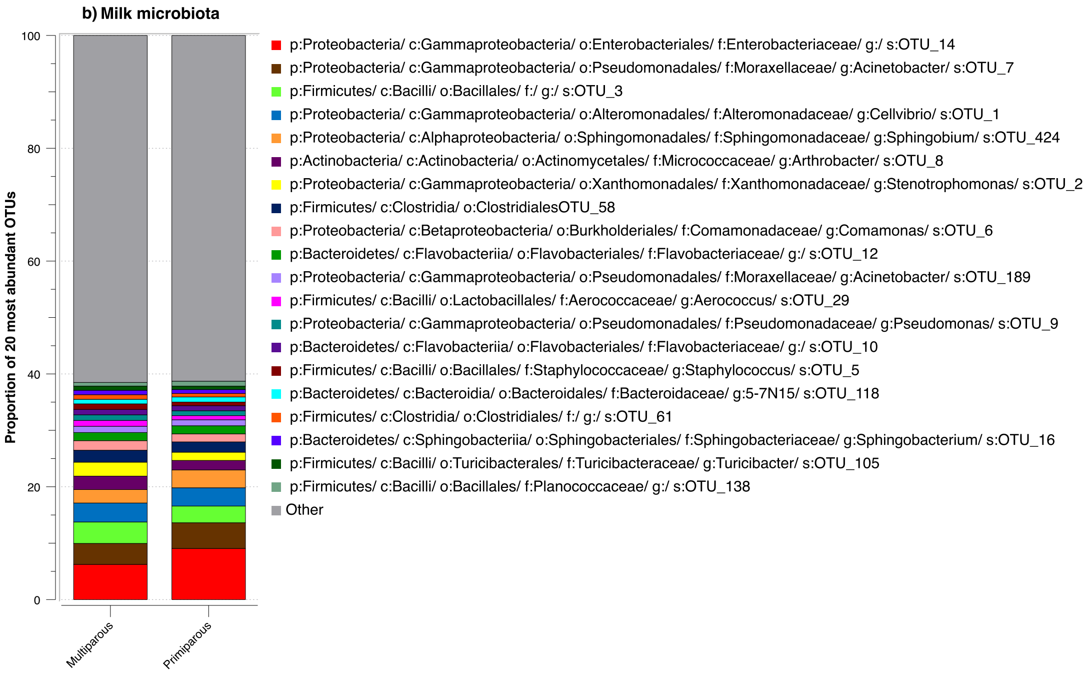

Figure S1. Niche-specific distribution of abundant OTUs. Stacked bar charts shows the proportions of the 20 most abundant non-random OTUs (present in at least 25% of the samples) within the a) teat canal and b) milk microbiota of udders with low SCC (< 200,000 cells/mL). After removing the OTUs that were not present in at least 25% of the samples, the OTU table was normalized to an even depth of 3000 OTUs per sample. Color codes denote the taxonomic classification of representative OTUs aligned against GreenGenes database at 97% similarity threshold.

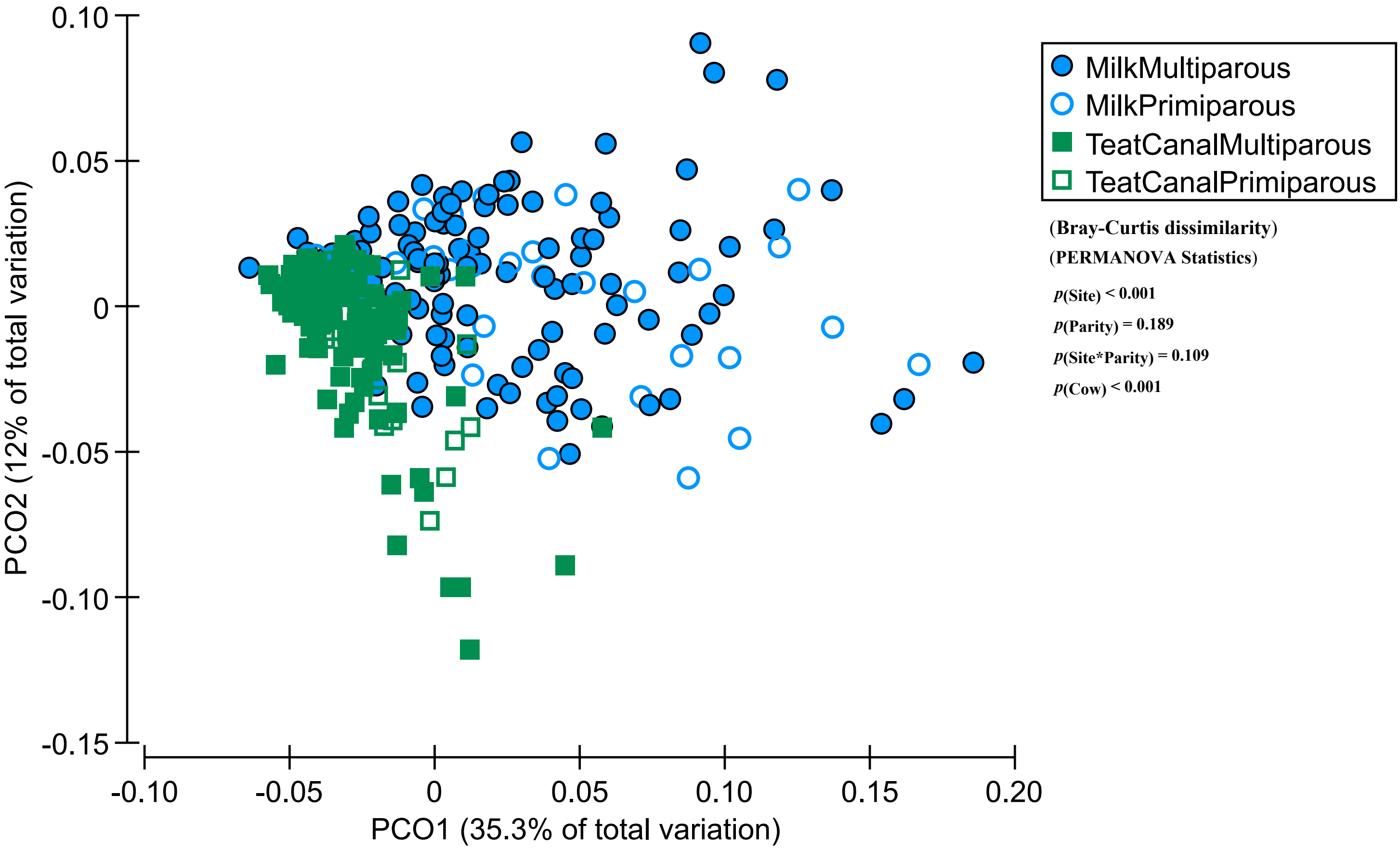

Figure S2. Comparison of β-diversity between teat canal and milk microbiota. Principal coordinate analysis (PCoA) was used for visualization of weighted UniFrac distances of the microbial communities. The OTU table was normalized using cumulative sum scaling (CSS) transformation. PERMANOVA was used to test for distinction of clustering patterns based on different niches of mammary gland and parity. The effect of cow was included as random factor in all comparison. For all comparisons, *p*-values < 0.05 were considered as significant.

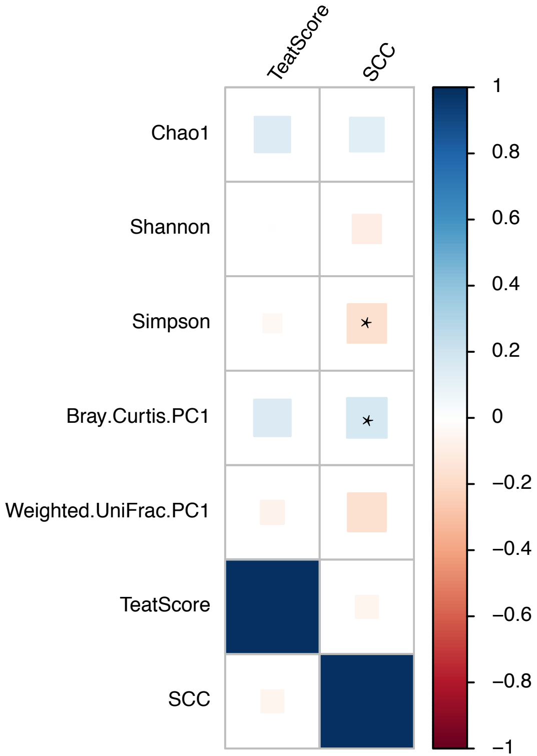

Figure S3. Relationships between udder health parameters and diversity metrics of the milk microbiota. Spearman's correlation coefficient was used to explore the relationships between SCC, teat end hyperkeratosis scores, and diversity metrics of the milk microbiota including community richness (Chao1 index of richness), α-diversity (Shannon's and Simpson's indices of diversity), β-diversity (Bray-Curtis dissimilarities and weighted UniFrac distances of microbial communities). "*" Indicates *p*-value < 0.05. The color ramp indicate the type and strength of the Spearman's correlation coefficient (rho): rho =1 showing strong positive correlation and rho = -1 showing strong negative correlation between the two parameters.

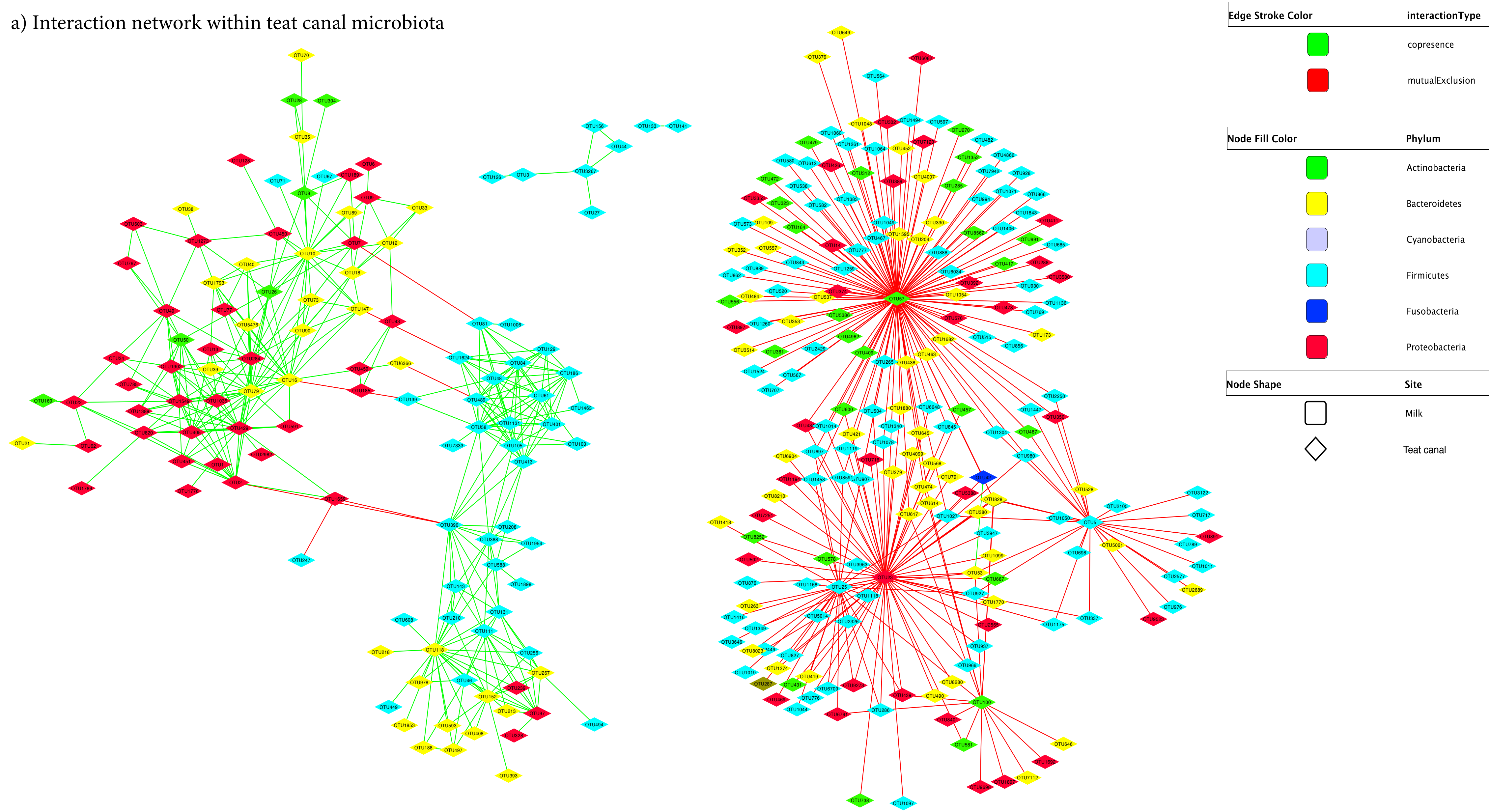
a) Interaction network within teat canal microbiota
Edge Stroke Color
interactionType
copresence
mutualExclusion
Node Fill Color
Phylum
Actinobacteria
Bacteroidetes
Cyanobacteria
Firmicutes
Fusobacteria
Proteobacteria
Node Shape
Site
Milk
Teat canal

b) Interaction network within milk microbiota

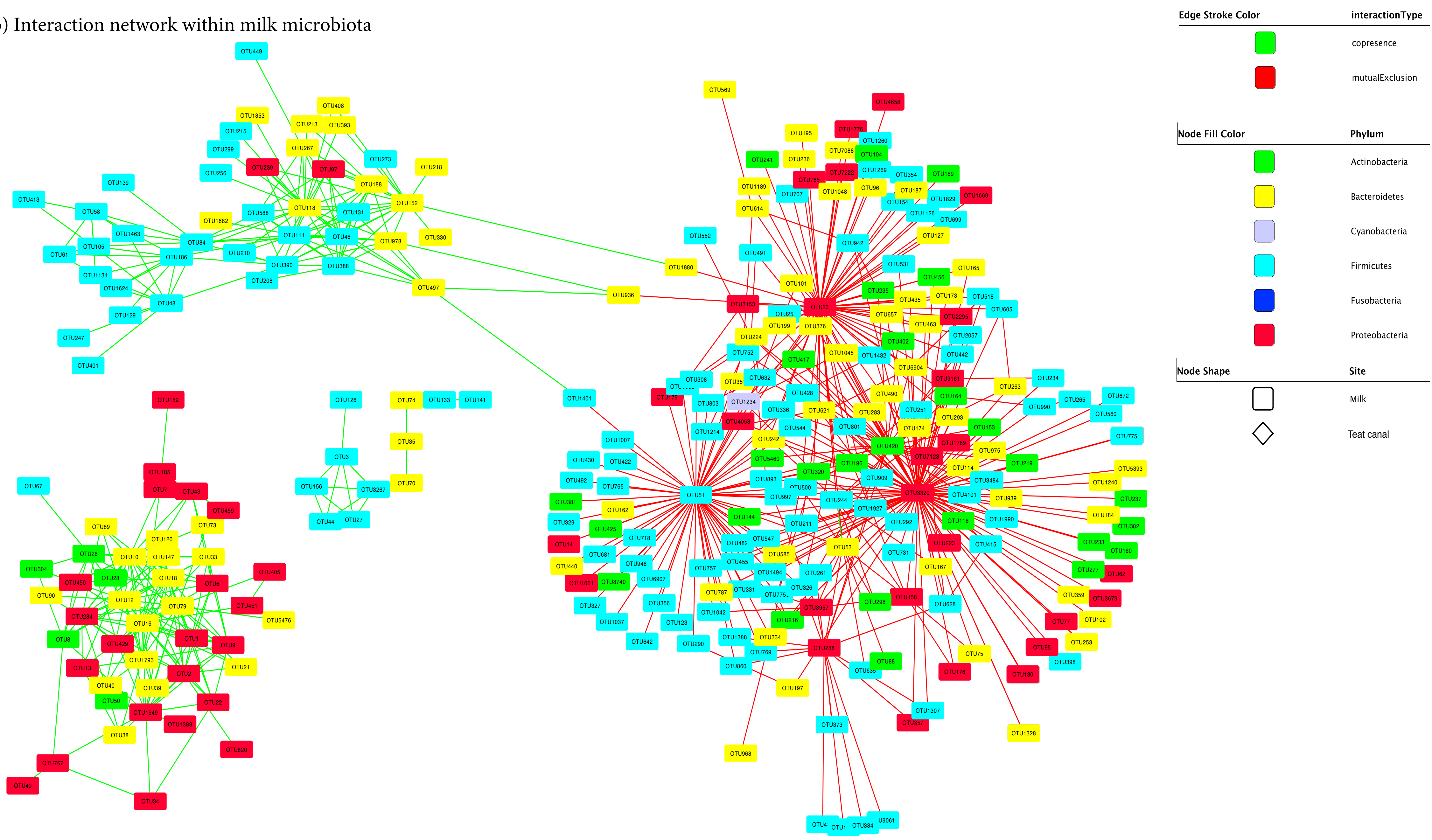

c) Interaction network between teat canal and milk microbiota

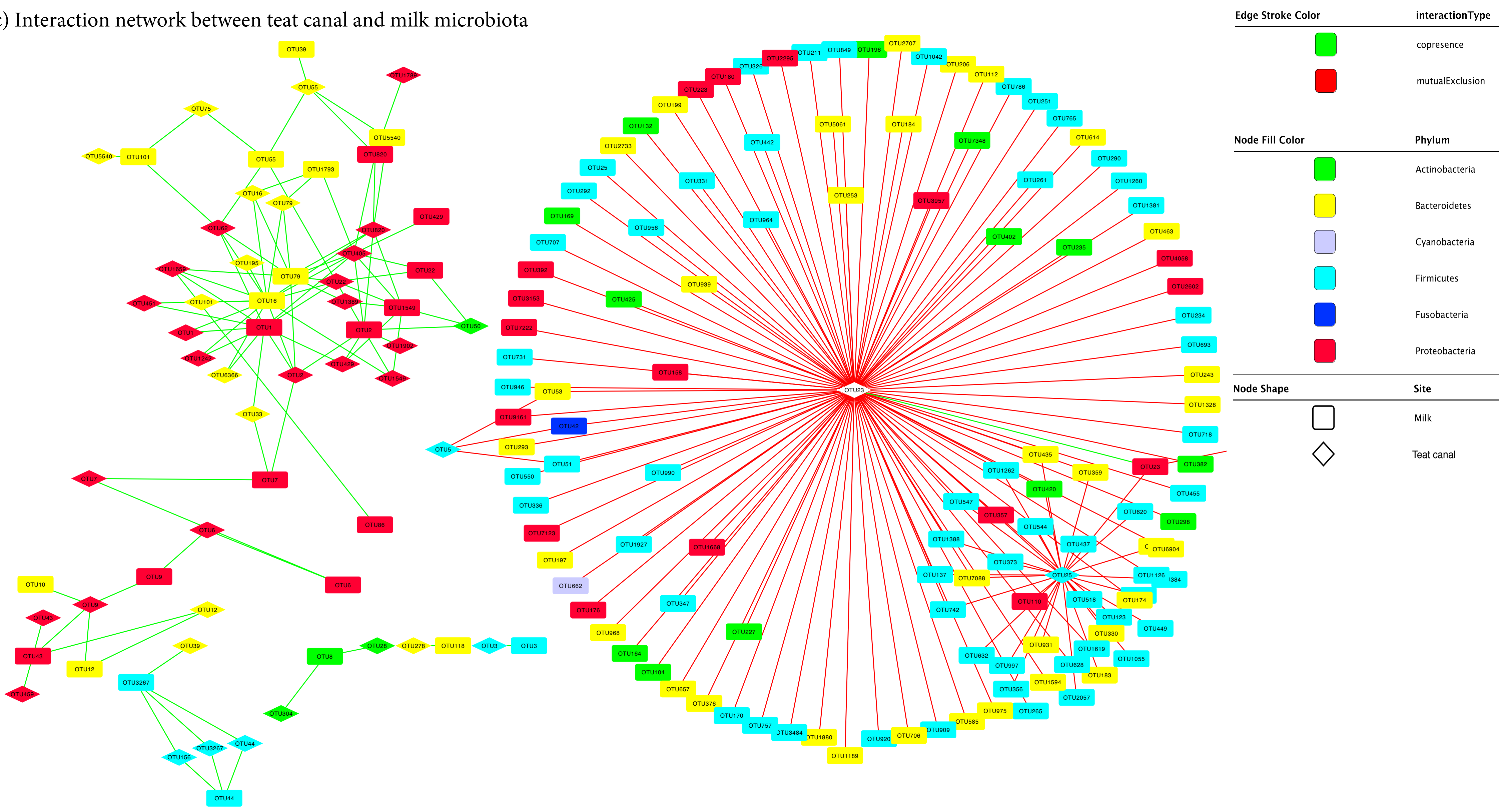

Figure S4. Bacterial co-occurrence and co-exclusion networks. Correlation network analysis (CoNet) was used to explore microbial co-occurrence/mutual-exclusion relationships. In this ensemble method, a combination of diverse measures of correlation (including Pearson's, Spearman's, and Kendall's correlation coefficients) and dissimilarity (Bray-Curtis, Kullback-Leibler, and Jensen Shannon dissimilarities) were used to overcome major challenges in the inference of co-occurrence and/or co-exclusion patterns. Networks show significant relationships (FDR corrected $q < 0.05$) among bacterial OTUs within a) teat canal microbiota, b) milk microbiota, and c) between teat canal and milk microbiota. Nodes represent representative OTUs, colored based on originating phyla, and edges represent significant co-occurrence/co-exclusion relationships, colored based on the type of relationships (red = negative relationship (mutual co-exclusion), and green = positive relationship (co-occurrence). The shapes of the nodes represents the origin of each representative OTU; diamonds for OTUs belonging to teat canal microbiota and rectangles for the OTUs belonging to milk microbiota.

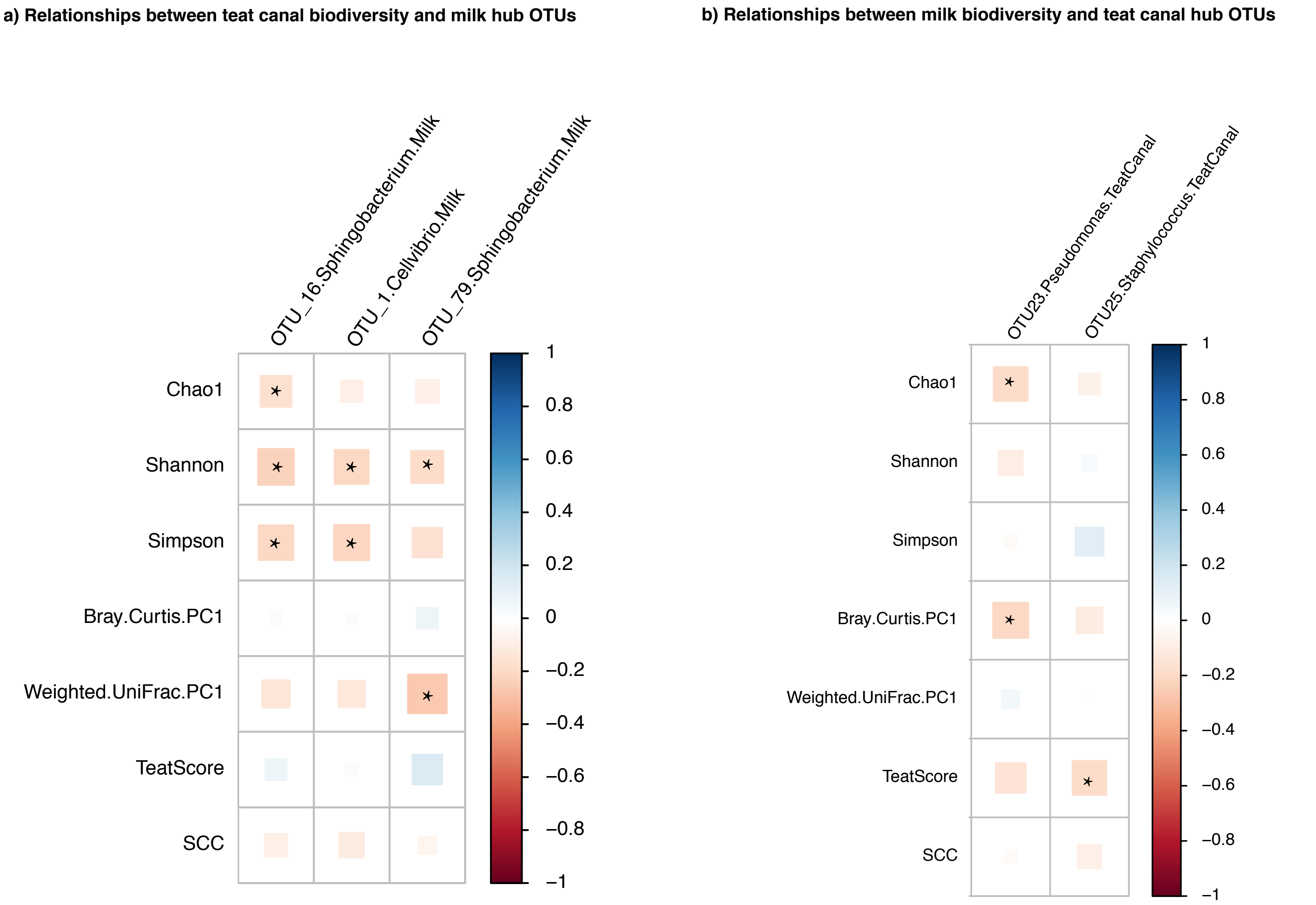

Figure S5. Between-niche relationships of hub OTUs with diversity metrics of microbiota and udder health parameters. Spearman's correlation coefficient was used to explore the relationships of "between-niche hub OTUs" (that are OTUs within a) milk, and b) teat canal microbiota that had the highest number of connections with the microbiota of teat canal and milk, respectively) with SCC, teat end hyperkeratosis scores, and diversity metrics including community richness (Chao1 index of richness), α-diversity (Shannon's and Simpson's indices of diversity), β-diversity (Bray-Curtis dissimilarities and weighted UniFrac distances of microbial communities). "*" Indicates *p*-value < 0.05. The color ramp indicate the type and strength of the Spearman's correlation coefficient (rho): rho =1 showing strong positive correlation and rho = -1 showing strong negative correlation between the two parameters.

a) Future incidences of clinical mastitis based on the distribution of hub OTUs

OUT ID	Taxonomic classification	% (number) of future incidences of clinical mastitis within high category	% (number) of future incidences of clinical mastitis within low category
OTU118	5-7N15 - Bacteroidaceae	53.33% (n = 8)	46.66% (n = 7)
OTU152	5-7N15 - Bacteroidaceae	40.00% (n = 6)	60.00% (n = 9)
OTU111	Phascolarctobacterium	**20.00% (n = 3)**	80.00% (n = 12)
OTU18	Sphingobacteriaceae	73.33% (n = 11)	26.66% (n = 4)
OTU16	Sphingobacterium	**80.00% (n = 12)**	20.00% (n = 3)
OTU79	Sphingobacterium	66.66% (n = 10)	33.33% (n = 5)

b) Relationships between candidate foundation OTUs and selected bacterial genera

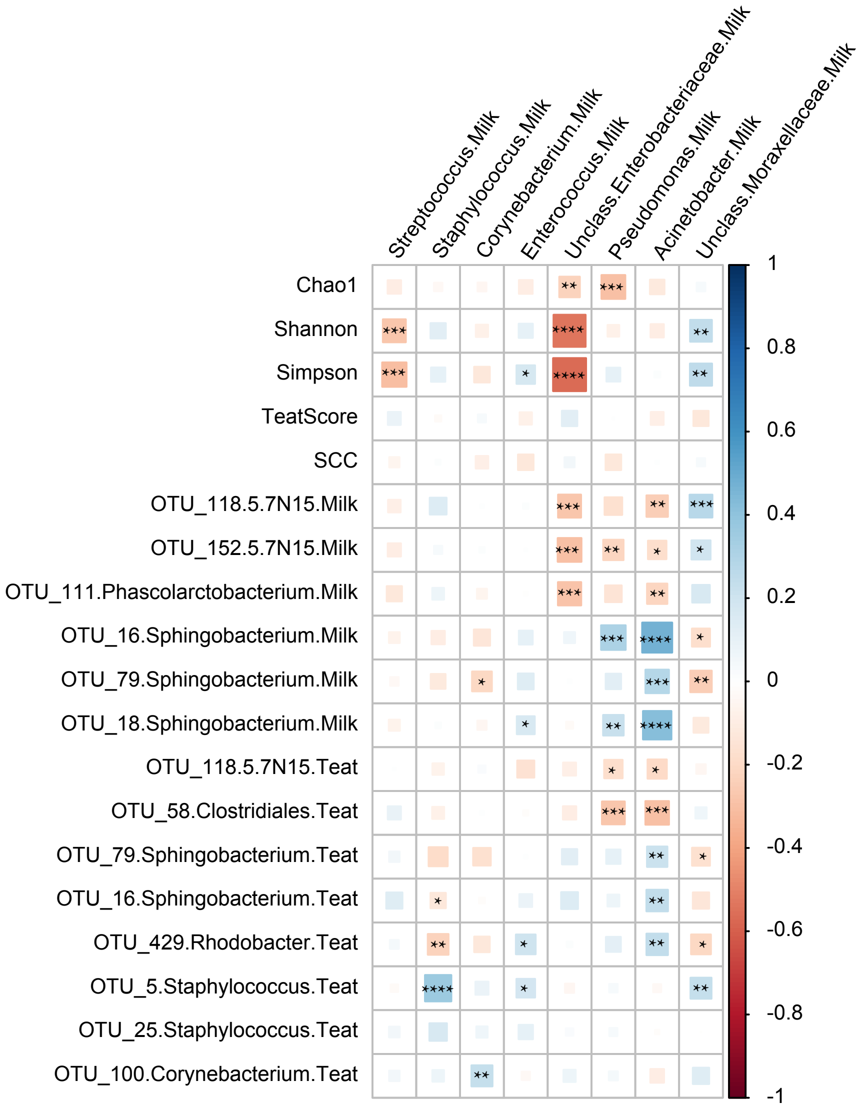

Figure S6. Association of hub species with future incidence of clinical mastitis and proportions of potentially pathogenic bacterial genera. a) Table shows the future incidences of clinical mastitis based on the distribution of hub OTUs within milk microbiota. Samples were categorized into two groups that either contained high (≥ 10 OTUs/4000 sequencing reads) or low (< 10 OTUs/4000 sequencing reads) number of the selected hub OTUs. Highlighted texts show highest (80%) and lowest (20%) incidence of clinical mastitis based on the distribution of hub OTUs. b) Relationships between candidate foundation OTUs and selected bacterial genera. Spearman's correlation coefficient was used to explore the relationships between the relative abundances of selected bacterial genera (genera that are known to contain major mastitis pathogen species) and the proportion of candidate foundation OTUs found within milk or teat canal microbiota, community richness (Chao1 index of richness), α-diversity (Shannon's and Simpson's indices of diversity), and udder health parameters including teat end hyperkeratosis scores and somatic cell counts (SCC) of the milk samples. "*" Indicates *p*-value < 0.05. "**" Indicates *p*-value < 0.01. "***" Indicates *p*-value < 0.001. "****" Indicates *p*-value < 0.0001. The color ramp and the size of the rectangles indicate the type and strength of the Spearman's correlation coefficient (rho): rho =1 showing strong positive correlation and rho = -1 showing strong negative correlation between the two parameters.

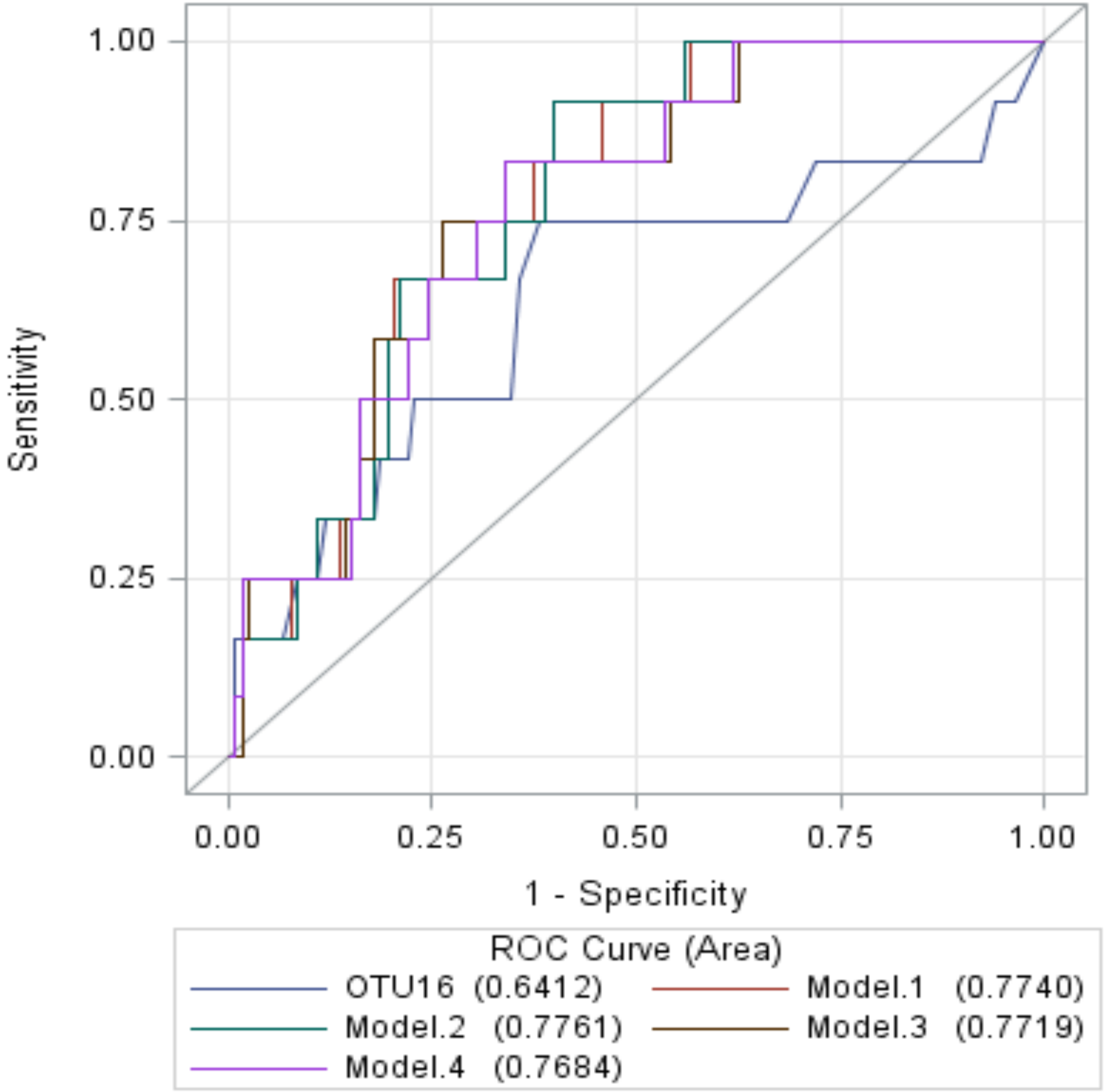

Figure S7. Discriminatory power of selected OTUs for prediction of mastitis susceptibility. Receiver operating characteristics (ROC) curves and area under the curve (AUC) values were used to assess the discriminatory power of the relative abundances of selected OTUs (foundation OTUs and/or OTUs that were found to be associated with the incidences of clinical mastitis during the 90-day post-sampling record keeping period) to predict susceptibility to clinical mastitis. Color codes represent the logistic regression models that were used for ROC analysis: "blue" denotes ROC based on the inclusion of OTU16 , "red" denotes model 1: based on the combination of OTU16, OTU6366, and OTU978, "green" model 2: based on the combination of OTU16, OTU6366, OTU111, and OTU978, "brown" model 3: based on the combination of OTU16, OTU6366, OTU79, and OTU978, and "purple" model 4: based on the combination of OTU16, OTU6366, OTU79, OTU978, and OTU 111. The straight line represents the null model.
